# What We Have Learned about Autism Spectrum Disorder from Valproic Acid

**DOI:** 10.1155/2013/712758

**Published:** 2013-12-17

**Authors:** Taylor Chomiak, Nathanael Turner, Bin Hu

**Affiliations:** Department of Clinical Neurosciences, Hotchkiss Brain Institute, Faculty of Medicine, University of Calgary, 3280 Hospital Drive NW, Calgary, AB, Canada T2N 4N1

## Abstract

Two recent epidemiological investigations in children exposed to valproic acid (VPA) treatment *in utero* have reported a significant risk associated with neurodevelopmental disorders and autism spectrum disorder (ASD) in particular. Parallel to this work, there is a growing body of animal research literature using VPA as an animal model of ASD. In this focused review we first summarize the epidemiological evidence linking VPA to ASD and then comment on two important neurobiological findings linking VPA to ASD clinicopathology, namely, accelerated or early brain overgrowth and hyperexcitable networks. Improving our understanding of how the drug VPA can alter early development of neurological systems will ultimately improve our understanding of ASD.

## 1. Introduction

The core clinicopathology of autism spectrum disorder (ASD) is currently thought to be characterized by accelerated or early brain tissue overgrowth in regions involved in emotional, social, and communication functions [1–6]. Although much of the earlier evidence regarding accelerated or early brain overgrowth was based on head circumference measurements which may not be as robust as previously thought [7] (but see [8]), more recent longitudinal brain imaging studies have provided further evidence of this abnormal pattern of brain growth in ASD [3, 6]. In fact, abnormal pathological overgrowth in ASD may even persist into adulthood in certain brain regions [9].

Exposure to exogenous chemicals during pregnancy can interfere with cortical development and the maturation of offspring. One such exogenous chemical is the short chain fatty acid valproic acid (VPA). VPA is a drug used in humans primarily for epilepsy and seizure control, although it has been used in many nonepileptic conditions as well [10]. Based on several case studies and small-scale population-based studies in humans [11–16], in addition to mounting experimental evidence in animals [17–32], VPA has known teratogenicity and has long been suspected as a risk factor for ASD. This year, however, both a prospective study and a large-scale population-based study were published providing the most substantial evidence to date linking prenatal VPA exposure to an increased risk of ASD [33, 34]. Thus, in this paper we will review epidemiological evidence linking VPA to ASD and will discuss two promising leads in the study of ASD in light of human clinical findings and the valproic acid animal model of ASD.

## 2. Valproic Acid and ASD: Epidemiological Evidence

Previous links between the antiepileptic drug VPA and ASD have been found and explored in several case studies and retrospective studies. VPA has long been known to cause physical malformations and developmental disabilities, features of the clinical entity termed fetal valproate syndrome (FVS). Laegreid and colleagues in a 1993 study examined seven children with FVS, two of which were also exposed to benzodiazepines. Two of the seven cases showed autistic traits, with 2 more showing marked developmental delays [16]. The next year Christianson et al. published a case study describing two sibling pairs, all having FVS to various degrees, with one having been diagnosed with infantile autism [11]. A second case of a boy with FVS and ASD was reported by Williams et al. in 1997, followed up with 5 more cases described by the same authors in 2001 [12, 13]. Several larger retrospective studies have also been published providing further support that *in utero* VPA exposure and fetal valproate syndrome may be linked to an increased risk of ASD, where, for example, it has been reported that the rate of ASD in the children of VPA-treated mothers may be roughly eight times larger than that of the general population [14, 15, 35]. Nevertheless, many of these studies had mentioned the need for future study to confirm the association between VPA and ASD.

A recently published prospective cohort study by Bromley et al. [33] explored the relationship between prenatal exposure to anti-epileptic drugs (including VPA) and the risk of neurodevelopmental disorders such as ASD, ADHD, and Dyspraxia. They performed an 11-year study, observing the physical and cognitive development of 415 children born to mothers with epilepsy and nonepileptic controls, with a final outcome of neurodevelopmental diagnosis by 6 years of age. Their results showed a significant increase in the risk of neurodevelopmental disorders in those women taking VPA during pregnancy, with autism being the most frequent diagnosis. The researchers also suspected a dose-dependent mechanism of VPA action but were not able to show it with significance in the study due to low numbers of mothers taking VPA. Although this study had a fairly small cohort and a relatively early endpoint (the average age of diagnosis of ASD in the UK is 5–11 years of age), it was the first prospective study to explore the relationship of VPA to ASD [33].

A much larger and detailed population-based study by Christensen et al. [34] that focused more on ASD than other neurodevelopmental disorders was also published this year. The large sample (655,615 children) and thorough control of the study provide the strongest evidence to date on the relationship between VPA and ASD. Data were from children born in Denmark from 1996 through 2006 over 14 years until the end of 2010. In their analysis they controlled for other known risk factors of ASD and potential confounders such as parental psychiatric disease, parental age at conception, congenital malformations, and maternal epilepsy. They divided the outcomes as Autism Spectrum Disorder (ASD) or childhood autism (the most severe diagnosis, simply referred to as “autism”) and reported absolute risks over the 14 years and hazard ratios (HR) for each. The absolute risks for all children studied for ASD and autism were 1.53% and 0.48%, respectively, whereas for those exposed to VPA *in utero,* the risks were 4.42% (HR 2.9) and 2.5% (HR 5.2). In fact, even when looking at all stratifications and controls, it was concluded that there was a significantly increased risk of children developing either ASD or autism in women taking VPA during pregnancy [34].

Accumulating epidemiological studies have shown an increased risk of children developing a neurodevelopmental disorder, and ASD in particular, if their mothers take VPA during pregnancy. These findings should encourage a discussion on the risks and benefits of the treatment during pregnancy and provide an opportunity to explore how this chemical can alter early developing biological systems that may relate to ASD in animal models. This may facilitate the discovery of other VPA-like substances that may subsequently prove to be environmental or nutritional risk factors for the development of ASD, and these could further elucidate the role of exogenous chemicals on ASD pathology. The authors of the two recent papers discussed above also noted a need to define the mechanism behind the increased risk of ASD with VPA exposure. Several questions arise as to the relationship between environmental exposure to chemicals such as VPA in the manifestation of ASD [33, 34]. Christensen et al. suggested several mechanisms by which VPA may increase the risk of ASD that need further study, including interference in neurotransmitter function, neuronal apoptosis or plasticity, histone deacetylase inhibition, and disruption of folic acid metabolism [34]. Studying the effects of VPA on biological tissue will provide many avenues to explore the possible mechanism(s) of ASD.

## 3. Valproic Acid Rodent Model of ASD

The model of VPA exposure proposed by Rodier et al. in 1996 is one of the most frequently studied animal models of ASD [36, 37] (also see [38] for review). As reviewed by Dufour-Rainfray et al., this model exhibits many of the structural and behavioural features that can be observed in ASD patients [39]. For example, rats exposed to VPA *in utero* can exhibit physical malformations (e.g., ear malformations) which have been compared to similar abnormalities that have been observed in some autistic patients (e.g., posterior rotation of the outer ear) [39]. Furthermore, several independent laboratories have also shown that prenatal VPA exposure can lead to behavioral abnormalities that are strikingly similar to those observed in autistic patients; including decreased social interactions and sensitivity to pain, increased sensitivity to nonpainful stimuli, repetitive/stereotypic-like activity, increased anxiety, abnormally high and longer lasting fear memories which are over-generalized and harder to extinguish, and changes in specific types of pup ultrasonic vocalizations [17–28, 30, 31, 39]. At the cellular level, recent morphological data has also suggested that a decrease in spine density in forebrain structures may be a substrate for distal hypo-connectivity, while the increase in dendritic length might support the enhancement of local hyperconnectivity [40]. As pointed out by the authors, this notion is consistent with the ‘‘intense world” theory of ASD which postulates a main neuropathology characterized by hyperfunctioning of local neural microcircuits [40–42].

An animal model, to be considered as a relevant model of a psychiatric condition described in humans, should fit several criteria usually described as construct, face, and predictive validity [43]. Although the effects of VPA have been tested in rodents for many years, only relatively recently it has been used to model ASD in rodents for studying ASD-like behavioural features and potential treatment interventions [1, 26]. Roullet et al. have noted that the VPA model exhibits both construct and face validity [38], and recent work has now provided evidence that it may also exhibit predictive validity [26, 44]. For example, Schneider et al. previously reported that environmental enrichment including multisensory stimulation reversed almost all behavioral alterations observed in pups exposed to VPA *in utero* [26], while Woo and Leon have recently shown in a randomized controlled trial that environmental enrichment in the form of multiple sensorimotor stimuli was also effective in ameliorating some of the symptoms in autistic children [44].

It has been suggested that methodological issues may have limited the effectiveness of utilizing the prenatal VPA rodent model to study ASD [45]. Interestingly, similar to the prenatal model, many ASD-like behavioural and pathological features have also been observed with VPA exposure during the early postnatal period (equivalent to the third trimester and perinatal period in humans) as previously shown by Yochum et al. [46–49]. This suggests a mechanism that is effective over different developmental time points and may offer an additional approach for studying ASD in rodents. Indeed, the postnatal VPA model may partly be explained by the clinical observation that the maternal serum VPA free-fraction increases in the third trimester and is highest at birth [50]. This is consistent with the idea that bioavailability may play an important role in VPA teratogenicity and that pre- and early postnatal VPA exposure may be quite useful in studying the effects of VPA to help elucidate neuropathological mechanisms of ASD [47]. For instance, VPA exposure during prenatal and early postnatal periods in rodents can lead to accelerated or early brain overgrowth that is reminiscent of that observed in humans. Prenatal VPA exposure on the one hand has been shown to induce a more generalized pathology and can induce macrocephaly in rat brain [51], while early postnatal VPA exposure, on the other hand, has been suggested to induce a more regionally selective pattern of aberrant overgrowth that may be particularly prominent in the temporal association cortex [49]. Thus, while it has been reported that the period of exposure to VPA that is likely to result in ASD is the first trimester of pregnancy, the possibility that ASD can also result from later effects should not be excluded [39].

## 4. Valproic Acid and Epigenetic Regulation 

**Figure 1:**
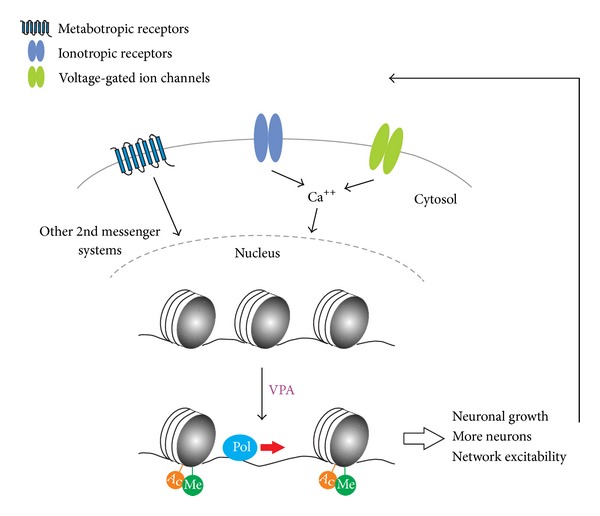
A schematic of some key molecular and cellular changes associated with VPA related to ASD neuropathology. Common clinicopathology associated with ASD is accelerated or early brain overgrowth and increased network excitability. Early or accelerated overgrowth may result from more synapses, increased denritic growth, and/or increased number of cells, while increased network excitability may result from hyperconnectivity and/or hyperplasticity of microcircuits, presumably driven by synaptic mechanisms (i.e., enhanced paired-pulse facilitation and long-term potentiation) that have been shown in rat brain both *in vitro* and *in vivo* following prenatal VPA exposure [56, 57]. All of these aspects are also likely regulated by histone acetylation (orange circles “Ac”) and/or histone methylation (green circles “Me”) and possibly DNA methylation (DNA methylation not shown). VPA has been shown to increase histone acetylation and histone methylation that can promote gene activation (symbolized by blue circle “Pol”; RNA polymerase). While VPA may disrupt the balance between excitatory and inhibitory neuronal activities through histone acetylation modulation [58], the role of histone methylation on VPA-related ASD neuropathology is much less clear. Increased connectivity and network excitability may further influence this process by activating metabotropic, ionotropic, and voltage-gated ion channels and subsequent intracellular signaling cascades.

Dysregulated biochemical pathways can have a profound impact on key cellular processes that may ultimately alter neuronal and network developmental trajectories. We know that the coordinated execution of gene programs involved in neural network maturation is highly regulated by epigenetic processes and that many biochemical pathways can converge to influence these processes [52–55] (Figure 1). Some of the mechanisms that may be involved include histone acetylation, histone methylation, and possibly DNA methylation.

Valproic acid is a commonly used antiepileptic drug [39]. Classically, the mechanism of action of VPA has focused on increases in brain concentrations of gamma-aminobutyric acid (GABA), the major inhibitory neurotransmitter [39], but has also included modulation of voltage-gated sodium channels and glutamatergic signaling [59]. However, it was previously shown in an animal model of seizure that VPA treatment did not significantly affect seizure strength or frequency for the first several days following induction but did, interestingly, protect the animals from seizure-induced cognitive impairment [60]. The authors concluded that their results could be explained, at least partially, by histone deacetylase (HDAC) inhibition [60]. In fact, several studies have since been published supporting the notion that VPA may block HDAC activity in neurons [52, 58, 61].

Histone deacetylases (HDACs) play an important role in regulating gene transcription and phenotype differentiation [53–55]. For example, MacDonald and Roskams reported specific expression patterns of HDAC1 and HDAC2 (both class I HDACs) in the murine brain at multiple developmental ages. HDAC1 is expressed in neural stem cells/progenitors and glia, while HDAC2 is upregulated in postmitotic neuroblasts and neurons but not in fully differentiated glia [62]. HDAC modulation in different cell types and at different maturational time points may therefore lead to dramatically different outcomes and may help to explain why HDAC inhibition in adulthood can improve ASD-like symptoms in rodents exposed to VPA *in utero* [63].

VPA is a nonselective HDAC inhibitor. Class I HDACs, which include HDAC1 and HDAC2, are expressed in the CNS, and VPA has the net effect of inhibiting their activity via different mechanisms. VPA inhibits HDAC1 through interaction with the enzyme's catalytic site, while VPA induces proteasomal degradation of HDAC2 [64, 65]. This is important as inhibition of class I HDACs can lead to increases in synapse numbers and a robust facilitation of excitatory synapse maturation [52]. Furthermore, VPA can also lead to increased neurite growth and promote neural proliferation [51, 66], both of which are thought to contribute to the accelerated or early overgrowth observed in ASD [1].

ASD structural pathology is regionally selective, exhibiting changes that are prominent in the prefrontal and temporal association cortices [1–3] that can even persist into adulthood [9]. These areas have been found to exhibit a slow and protracted endogenous pattern of development under normal conditions, remaining relatively immature well into postnatal life [67–72]. This pattern of maturation appears to be needed for appropriate cognitive development and allowing for the maturation and stabilization of lower-order networks with which to build upon [73–75]. However, the protracted maturation pattern of these prefrontal and temporal areas may confer an enhanced vulnerability to HDAC inhibition compared to networks that have already attained full maturity [1]. This vulnerability may lead to prominent increases in the structural assembly of synapses and growth of neural networks, both of which are thought to contribute to the underlying clinicopathology of ASD [1, 76].

Histone methylation is another important epigenetic mechanism related to histone acetylation that can regulate gene transcription, developmental gene programs, and cellular phenotype [55, 77, 78]. Much like that of histone acetylation, it has been proposed that histone methylation may be reversible, dynamic, and also important in the teratogenicity of VPA [78]. For example, Tung and Winn reported that not only did *in utero* exposure to VPA lead to HDAC inhibition and increased histone acetylation of mouse fetal tissue, but it was also associated with changes in histone methylation [78]. In fact, this study found significant increases in di- and trimethylated histone 3-K4 and significant decreases in monomethylated histone 3-K9, which may represent sites involved in gene activation and repression, respectively [77]. Indeed, these epigenetic changes are likely involved in the multiple genes that can be influenced by early VPA exposure [58].

Adding another layer of complexity to the epigenetic control of gene regulation is DNA methylation, where methyl groups covalently bound to the 5′ position of cytosine project into the major groove of DNA, thus inhibiting the binding of transcription factors [78]. While there is interplay between DNA methylation and histone acetylation [79], somewhat surprisingly, the significance of DNA methylation as a mechanism of VPA-induced teratogenesis remains debatable [78]. Thus, the role VPA plays on DNA methylation and the interplay between DNA methylation and histone acetylation will be an important area for future research.

## 5. Valproic Acid, Overconnectivity, and Hyperexcitable Networks

Clinically apparent seizures are not uncommon in ASD [80]. While there has been a long standing association between ASD and seizure, even children that do not present with clinical seizure can exhibit subclinical seizure and epileptiform electroencephalographic (EEG) abnormalities [80, 81]. Thus, it is postulated that accelerated or early brain overgrowth is accompanied by pathological changes in network excitability.

The rodent VPA model of ASD allows for intrusive electrophysiological study of living tissue unavailable in humans to uncover the nature and potential causes of these suspected electrophysiological abnormalities. One prevailing theory of ASD, the “intense world” theory [41, 42], has been refined through the study of the VPA rat model and postulates that several areas of the brain including the prefrontal cortex and amygdala display hyperreactivity of the microcircuitry of pyramidal cells compared to controls [22, 23, 41, 42, 56, 57]. The amygdala bares particular relevance, for example, as it has been functionally related to ASD due to its role in socioemotional behaviour and can also exhibit enlargement and hyperactivation in autistic children [82, 83]. Markram's group has shown enhanced fear memories, enhanced fear generalization, and impaired fear extinction in prenatally exposed VPA treated rats, along with the other ASD-like behavioural phenotypes [23]. As well, electrophysiological data from lateral amygdaloid nucleus slices showed increased reactivity to stimulation, increased long-term potentiation, and impaired inhibition [23]. A similar result was found by Lin et al. as they also reported hyperexcitability and enhanced LTP in amygdala lateral nucleus pyramidal neurons following prenatal exposure to VPA [17]. These authors stated that the increased ratio of synaptic excitation/inhibition in the amygdala might be associated with the characteristic behaviour in this ASD model [17], which was reiterated by Kim et al. based on their own work showing increased expression of glutamatergic proteins in prefrontal networks of postnatal brains of rat offspring exposed to VPA *in utero* [84]. Finally, as impairments in the GABAergic system may critically contribute to an increased synaptic excitation/inhibition ratio, an additional mechanism may also involve reduced GABAergic inhibition as recently shown in the temporal cortex of rat pups following prenatal VPA exposure [85].

Although functional imaging studies of the autistic brain have long suggested decreased activity, these recent findings from the VPA model propose an alternate explanation wherein electrophysiological abnormalities may cause network hyperactivity within hyperconnected microcircuits [22, 42, 57]. Indeed, a recent imaging study has shown hyperconnectivity in several large-scale brain networks of children with ASD [86]. While previous functional magnetic resonance imaging (fMRI) studies have been somewhat contradictory, these recent findings suggest that further research into brain network connectivity may lead to a better understanding of the pathology of ASD in the brain [86].

Furthermore, prenatal VPA exposure has been shown to increase NMDA receptor NR2A and NR2B subunits [22], with NR2A being important in epileptogenesis [87]. Consequently, this enhanced activity may subsequently lead to greater activity-dependent processes involved in controlling structural aspects neuronal and synaptic network maturation and growth (Figure 1). For example, it has been shown that activity-induced NR2A activation can lead to increased brain-derived neurotrophic factor (BDNF) expression [87]. Thus, given that BDNF is important in neuronal development [88–90] and epileptogenesis [91], this may be one of several signaling pathways involved in the clinicopathology of ASD.

While activity modulates not only structural aspects of neuronal connectivity such as dendritic branching and spine formation, it can also influence the neurochemical landscape of the neural network. For example, an emerging body of the literature is now providing mechanistic insight into neurotransmitter specification and the role of electrical activity in the developing nervous system [92–94]. Consequently, alterations in the proportions of excitatory and inhibitory neural transmitter phenotypes may play a very important role in the pathophysiology associated with ASD [95]. A number of neurodevelopmental processes ranging from early events of cell proliferation and differentiation, to late events involving maturation of the dendritic arbors and synapses, can all be influenced by activity [92, 94]. Thus, further study on the electrophysiological changes associated with rodent brains exposed to VPA is likely to uncover convergent molecular pathway(s) that drive these changes.

## 6. Conclusion

Based on accumulating clinical data, it is becoming increasingly clear that VPA is an important risk factor associated with ASD. In addition, a growing body of animal literature is also showing that VPA can lead to ASD-like features in rodents. Moreover, published work suggests that the VPA model of ASD appears to exhibit all elements of a relevant model, namely, construct, face, and predictive validity, and therefore is likely to prove useful in elucidating mechanisms of ASD clinicopathology. It is also important to note that the significance of the two recent epidemiological studies discussed in this paper may also lie in the potential of revealing VPA-like environmental or nutritional substances that could increase the risk of ASD. For example, it was recently shown that in certain cell types global histone acetylation and HDAC activity could be regulated by metabolites of intermediate metabolism [96]. Based on studies primarily in nonneuronal cells, it is also now evident that other agents, including those in the human diet, can be converted by metabolism to intermediates that can influence HDAC activity [96, 97]. Thus, improving our understanding of how VPA and VPA-like compounds can alter early nervous system development will ultimately improve our understanding of ASD.
